# Fish Tank Granuloma Caused by *Mycobacterium marinum*

**DOI:** 10.1371/journal.pone.0041296

**Published:** 2012-07-20

**Authors:** Ting-Shu Wu, Cheng-Hsun Chiu, Chih-Hsun Yang, Hsieh-Shong Leu, Ching-Tai Huang, Yi-Chieh Chen, Tsu-Lan Wu, Pi-Yueh Chang, Lin-Hui Su, An-Jing Kuo, Ju-Hsin Chia, Chia-Chen Lu, Hsin-Chih Lai

**Affiliations:** 1 Division of Infectious Diseases, Department of Internal Medicine, Chang Gung Memorial Hospital, Chang Gung University College of Medicine, Taoyuan, Taiwan; 2 Graduate Institute of Clinical Medical Sciences, Chang Gung University, Taoyuan, Taiwan; 3 Division of Infectious Diseases, Department of Pediatrics, Chang Gung Children's Hospital, Chang Gung Memorial Hospital, Chang Gung University College of Medicine, Taoyuan, Taiwan; 4 Department of Dermatology, Chang Gung Memorial Hospital, Chang Gung, Taoyuan, Taiwan; 5 Department of Plastic and Reconstructive Surgery, Chang Gung Memorial Hospital, Chang Gung University College of Medicine, Taoyuan, Taiwan; 6 Department of Laboratory Medicine, Chang Gung Memorial Hospital, Chang Gung University College of Medicine, Taoyuan, Taiwan; 7 Department of Medical Biotechnology and Laboratory Sciences, Chang Gung University, Taoyuan, Taiwan; 8 Graduate Institute of Medical Biotechnology and Laboratory Sciences, Chang Gung University, Taoyuan, Taiwan; 9 Department of Respiratory Therapy, Fu-Jen Catholic University, New Taipei, Taiwan; 10 Research Center for Pathogenic Bacteria, Chang Gung University, Taoyuan, Taiwan; University of California Merced, United States of America

## Abstract

**Introduction:**

*Mycobacterium marinum* causes skin and soft tissue, bone and joint, and rare disseminated infections. In this study, we aimed to investigate the relationship between treatment outcome and antimicrobial susceptibility patterns. A total of 27 patients with *M. marinum* infections were enrolled.

**Methods:**

Data on clinical characteristics and therapeutic methods were collected and analyzed. We also determined the minimum inhibitory concentrations of 7 antibiotics against 30 isolates from these patients.

**Results:**

Twenty-seven patients received antimycobacterial agents with or without surgical debridement. Eighteen patients were cured, 8 failed to respond to treatment, and one was lost to follow-up. The duration of clarithromycin (147 vs. 28; *p* = 0.0297), and rifampicin (201 vs. 91; *p* = 0.0266) treatment in the cured patients was longer than that in the others. Surgical debridement was performed in 10 out of the 18 cured patients, and in 1 of another group (*p* = 0.0417). All the 30 isolates were susceptible to clarithromycin, amikacin, and linezolid; 29 (96.7%) were susceptible to ethambutol; 28 (93.3%) were susceptible to sulfamethoxazole; and 26 (86.7%) were susceptible to rifampicin. However, only 1 (3.3%) isolate was susceptible to doxycycline.

**Discussion:**

Early diagnosis of the infection and appropriate antimicrobial therapy with surgical debridement are the mainstays of successful treatment. Clarithromycin and rifampin are supposed to be more effective agents.

## Introduction

*Mycobacterium marinum* is a Runyon group I, slow-growing mycobacterium with an optimal growth temperature of 30°C [1]. It most frequently causes skin and soft tissue infections in the extremities [2]. Patients typically show clusters of nodules, ulcers, or verrucous plaques that may centripetally spread from the arms or legs in a sporotrichoid pattern; however, pulmonary infections, osteomyelitis, arthritis, and disseminated diseases are encountered to a lesser extent [3]–[6]. Many factors play important roles in causing *M. marinum* infections. These include prescription of local or systemic steroids or immunosuppressive agents, and structural lung diseases. Among these, the primary risk factor is exposure to aquatic environments or marine animals. Thus, *M. marinum* infection is also known as “fish tank granuloma” [7].

Currently, the most common regimens used for treating *M. marinum* infections are tetracycline, doxycycline (DOX), clarithromycin (CLR), and rifampicin (RIF) plus ethambutol (EMB) [8]–[10]. However, there are no standard regimens available for treating *M. marinum* infections; in addition, some unsatisfactory results were encountered with the available regimens [9]–[11]. The role of surgery is still a controversial issue [12]. So far, only 1 study has discussed the relationship between antimycobacterial agent selection and clinical outcomes on the basis of susceptibility testing results [9]. The aim of our study was to try to find the favorable therapeutic agents for *M. marinum* infection, evaluate the treatment benefits, and analyze factors influencing the treatment outcome.

## Methods

### Ethics statement

The study was approved by the Institutional Review Board of the Chang Gung Memorial Hospital Linkou Medical Center in December, 2009. Patients were requested to give written informed consent to store and use the data. No linkage of these data with other sources was done. No patient identifiers were included in the dataset used for this analysis. All bacterial strains were obtained from the Bacteria Bank, Department of Laboratory Medicine, Chang Gung Memorial Hospital.

### Clinical review, case definition, and classification of outcomes

A subject was defined as a patient if he/she had a culture-positive *M. marinum* infection. We studied the records of 27 patients who were enrolled for treatment at at the Chang Gung Memorial Hospital, Taiwan between January 1, 1999 and December 31, 2010. We retrospectively analyze the patients' medical charts for demographic data, contact history, infection sites, comorbidities, histological results, antimycobacterial regimens, surgical history, and outcomes. Among the 27 patients, 24 had either skin or soft-tissue infections, 1 had arthritis, 1 had a corneal infection, and 1 had a pulmonary infection. Twenty-six patients received antimycobacterial therapy, and 1 patient received steroid inhalation therapy, because this patient was treated as hypersensitivity pneumonitis; 10 of these 27 patients underwent at least 1 episode of surgical debridement. The antimycobacterial regimens included the following: RIF 600 mg/day (for pediatric patients, 10–15 mg/kg), EMB 1200 mg/day (for pediatric patients, 15–25 mg/kg), CLR 500 mg twice a day, DOX 100 mg twice a day, co-trimoxazole (sulfamethoxazole-trimethoprim) (SXT) 800/160 mg twice a day, and amikacin (AMK) 7.5 mg/kg twice a day. The clinical outcomes were classified as follows: (i) successful: remission of lesions without any sequelae; (ii) unsuccessful: persistent symptoms and signs of infection, relapse, or recurrence of infection within 6 months after completion of therapy, or who was lost to follow-up.

### Bacterial strains and chemicals

A total of 30 *M. marinum* isolates were collected. We isolated 3 strains from 1 patient, 2 strains from another, and 1 strain each from the remaining 25 patients. Bacteria were identified by traditional culture and biochemical methods [13]. The *hsp65* gene polymorphism analysis was performed to confirm the identification of *M. marinum*, as described by Telenti et al. [14]. All bacterial isolates were routinely maintained in skim milk collection tubes containing 50% glycerol and were refrigerated at −70°C before subcultivation. The reference strain *M. marinum* ATCC 927 was purchased from the American Type Cell Collection organization.

### Acid-fast staining (AFS)

We followed the procedure described by Kent and Kubica [15]. Briefly, the specimens were stained with carbolfuchsin, decolorized by 3% acid-alcohol, and finally counter-stained with methylene blue. Results of the AFS smears were reported as follows: 1+, if 1–9 acid-fast bacilli (AFB) were observed per 100 oil power fields; 2+, if 1–9 AFB were observed per 10 oil power fields; 3+, if 1–9 AFB were observed per oil power field; and 4+, if ≥10 AFB were observed per oil power field.

### Drug susceptibility test

The minimal inhibitory concentrations (MIC) of several antibiotics against *M. marinum* were studied by a microdilution assay, according to the methods described by Wallace and Brown et al. [16], [17]. In brief, the drugs were 2-fold serially diluted in 7H9 broth with OADC enrichment. Each well of the 96-well plate contained 10^−4^ L drug-containing broth. The final bacterial inocula had concentrations between 10^6^ and 10^7^ CFU/L. A 10^−5^ L bacterial suspension in Middlebrook solution that contained between 10 and 10^2^ CFU was dispensed into each well, including the negative control wells. The following antibiotics were tested: RIF, EMB, CLR, AMK, sulfamethoxazole (SMX), linezolid (LZD), and DOX. CLR was provided by the Pharmaceutical Products Division, Abbott Laboratories, North Chicago, Ill., USA, and LZD was provided by Pfizer Inc., New York, N.Y., USA. The other antimicrobials were purchased from Sigma Chemical Co., St. Louis, Mo., USA. The MIC was determined as the antibiotic concentration of the last well that showed no microbial growth from a series of dilutions of the antibiotic in a 96-well plate. The exception was SMX, for which the end-point was the well with approximately 80% growth inhibition compared with the growth in the negative control well. Susceptibility and resistance breakpoints of *M. marinum* strains were determined according to the Clinical and Laboratory Standards Institute (CLSI) standards [18]. Drugs susceptibility was defined at the following concentrations: RIF≤1 mg/L, EMB≤5 mg/L, CLR≤16 mg/L, AMK≤32 mg/L, SMX≤32 mg/L, and DOX≤4 mg/L. The breakpoint of LZD against mycobacteria was proposed as ≤8 mg/L, as described by Rodríguez et al. [19].

### Statistical analysis

Statistical analyses were performed using STATA version 11 (STATA Corporation, College Station, TX, USA). The Mann–Whitney *U* test was used for inter-group comparisons of continuous variables. Categorical variables were compared using the χ^2^ test or Fisher's exact test as appropriate. A p-value of <0.05 was considered statistically significant.

## Results

### Demogaphic data and clinical characteristics

In this study, we enrolled 27 patients who showed culture-positive *M. marinum* infections. According to the previously described criteria for clinical outcomes, 18 patients' outcomes were defined as successful, and 9 were unsuccessful. As shown in table 1, in the successful group, the median age was 48 years (inter-quartiles ranges [IQR], 35–64 years). In the unsuccessful group, the median age was 62 years (IQR, 41–65 years). Of the 27 patients, 11 were women, and 16 were men. These patients had underlying diseases such as hypertension (HTN) (5), coronary arterial disease (CAD) (2), cardiac dysrhythmia (1), diabetes mellitus (DM) (5), and gout (3). Twelve patients were exposed to a fish tank or pursued fishing as a hobby; 2 patients were exposed to shrimps and 1 had played in a swimming pool. One patient had received an intra-articular steroid injection for osteoarthritis. Three patients had minor or superficial trauma on their extremities. Eight patients had no contact history. The median (IQR) period from the appearance of lesions to visiting a doctor was 3 (1–6) months. Two patients had no documentation of this period. Fifteen patients had skin and soft tissue lesions on the right extremity, and 11 patients had lesions on the left side. Twenty-two patients had lesions on the upper limb, while only 4 patients had lesions on the lower limb. One of the patients showed lesions on more than 1 limb.

**Table 1:** Demographic data and treatment of *Mycobacterium marinum* infected patients.

Characters	Successful (n = 18)	Unsuccessful (n = 9)	P value
**Median age** [years (IQR)]	48 (35–64)	62 (41–65)	0.6431**a**
**Gender** [female: male]	6: 12	5: 4	0.2770**a**
**Antimicrobial agents** [median days (IQR); n]**c**			
INH	191 (73–226); 7	91; 1	0.5102**a**
RIF	201 (154–247); 13	91 (21–134); 5	0.0266**a**
EMB	220 (154–247); 13	91; 1	0.1724**a**
DOX	50.5 (28–73); 3	18 (12–21); 5	0.0507**a**
SXT	52.5 (28–77); 2	16 (14–18); 2	0.1213**a**
CIP	70.5 (22–119); 2	21 (15–143); 3	0.5637**a**
CLR	147 (91–281); 11	28 (21–35); 2	0.0297**a**
E		134; 1	
**Surgical debridement** [n]	10	1	0.0417**b**

IQR: inter-quartiles ranges, INH: isoniazid, RIF: rifampicin, EMB: ethambutol, DOX: doxycycline, SXT: sulfamethoxazole-trimethoprim, CIP: ciprofloxacin, CLR: clarithromycin, E: erythromycin.

a ) by Mann-Whitney *U* test.

b ) by Fisher's exact test.

c ) patients received one or more than one agent.

One patient had a right corneal lesion after a hook injury when finshing shrimps, and another patient had a rare lung infection during combination ribavirin and pegylated interferon therapy for her chronic C hepatitis.

**Figure 1:**
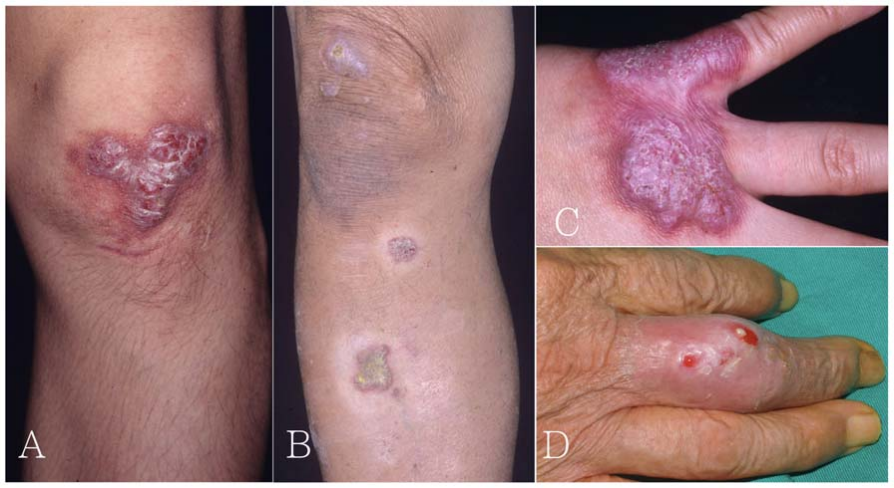
Cutaneous manifestations of *Mycobacterium marinum* infections. Footnote: *M. marinum* skin infections presented with (A) a solitary, or (B) multiple violaceous plaques with hyperkeratotic surface on limbs. The disease also presented as (C) a warty, verrucous plaque with an irregular border on hand, or (D) erythematous swelling of finger with pus discharge.

These patients did not consult a doctor until the symptoms worsened; the duration from the onset of symptoms to visiting a doctor varied from 15 days to as long as 3 years. The most common presentations were tender, erythematous nodules or plaques (18/27). Seven of the 27 patients presented with ulcerative wounds with a purulent discharge (figure 1). One of these patients had corneal involvement and complained of right-eye pain and photophobia, and another patient had dyspnea.

### Acid-fast staining and histopathological findings

Of the 27 acid-fast stained pus and tissue specimens, 9 (33.3%) were positive. Of these 5, 3, and 1 specimens scored 1+, 2+, 3+, respectively; 3 specimens had no AFS documentation. Samples from 23 patients were subjected to both histopathological examinations and bacterial culturing, while samples from 4 other patients underwent only culture. The most commonly documented pathological condition was suppurative granulomatous inflammation (8/23), followed by granulomatous inflammation (7/23), caseating granulomatous inflammation (2/23), and necrotizing granulomatous inflammation (2/23). Four specimens were reported to indicate chronic inflammation, rheumatoid nodule, focal alveolar damage, and corneal ulcer.

### Antimicrobial susceptibilities

The quality control strain (*M. marinum* ATCC 927) was susceptible to all 7 drugs. The results of *in vitro* susceptibility testing indicated that *M. marinum* isolates were basically sensitive to the drugs used in the test, with a sensitivity rate ranging between 86.7% (RIF) to 100% (CLR, AMK, and LZD); the only exception was DOX, to which only 1 (3.3%) isolate was sensitive (table 2).

**Table 2:** *In vitro* susceptibilities of 30 *M. marinum* isolates.

Antimicrobial agents	MIC 50	MIC 90	Range	Modal MIC	Geometric mean MIC	95% CI	Sensitive rate (%)
Rifampicin	1	2	0.125–4	1	0.7	0.5–1	86.7
Ethambutol	0.25	4	0.25–16	0.5	1	0.6–1.5	96.7
Clarithromycin	4	6	0.125–8	4	3	2.2–4.1	100
Amikacin	2	8	0.125–16	8	2.3	1.4–3.9	100
Sulfamethoxazole	4	32	0.5–64	4	4.7	2.7–8.1	93.3
Linezolid	2	4	0.5–4	2	1.7	1.4–2.2	100
Doxycycline	16	32	4–32	16	14.9	12.0–18.6	3.3

MIC: minimum inhibitory concentrations, CI: confidence interval.

### Treatment and outcome

Among these 27 patients, 25 patients got *M. marinum* skin and soft tissue infections, one had pulmonary infection, and the last had corneal infection. After medical treatment and/or surgical debridement, 18 were cured without any sequelae, 7 remained draining and unhealed wounds, 1 had complication of hypersensitivity pneumonitis, and one had right eye blindness.

For antimicrobial therapy, the median (IQR) duration of prescription (in days) for each antibiotic in the successful versus unsuccessful groups was as follows: CLR, 147 (91–281) vs. 28 (21–35) (*p* = 0.0297); and RIF, 201 (154–247) vs. 91 (21–134) (*p* = 0.0266). These figures indicate that the duration of prescription of CLR, and RIF was longer in the successful group than in the unsuccessful group. On the other hand, the median (IQR) duration of prescription for DOX was 50.5 (28–73) vs. 18 (12–21) (*p* = 0.0507) in the successful versus unsuccessful group , respectively. This indicated there is a tendency to treatment failure for prescription of doxycycline. Furthermore, the median (IQR) duration of prescription of the following antibiotics in the successful versus unsuccessful groups was as follows: isoniazid (INH), 191 (73–226) vs. 91 (*p* = 0.5102); EMB, 220 (154–247) vs. 91 (*p* = 0.1724); SXT, 52.5 (28–77) vs. 16 (14–18) (*p* = 0.1213); ciprofloxacin (CIP), 70.5 (22–119) vs. 21 (15–143) (*p* = 0.5637); and erythromycin, 134, respectively. Besides, 10 patients in the successful group had received surgical debridement; whereas, only 1 patient in the unsuccessful group had received surgical debridement (Fisher's exact test; *p* = 0.0417).

Eight patients received DOX treatment; of these, only 3 (37.5%) had successful results (table 3). In addition to the DOX therapy, 2 of these 3 patients further received adjuvant surgical debridement of the lesions, and the third patient received a combination of RIF and EMB treatment for 73 days, and consecutive prescription with CLR, RIF, and EMB for 147 days. Of the 5 patients who underwent unsuccessful DOX treatment, 1 received only local CIP treatment for corneal infection with sequela of right eye blindness, 1 had an overly short CLR and CIP treatment duration (21 days) with persistent nodules on her right hand,1 had an overly short RIF (21 days) and SXT (14 days) treatment duration with persistent plaques on his right hand, 1 further received SXT, CLR, CIP, and RIF, but had persistent nodules and relapsed 4 months after discontinuing the antibiotics, and the fifth unsuccessful patient became lost to follow-up.

**Table 3:** Clincal manifestations of *M. marinum* infected patients who received doxycycline therapy.

Case	Age	Gender	Infection source	Site of lesions	Histology	Antibiotics (days)	Surgery	Outcome
1	41	M	Shrimp sting	Cornea	Ulcer	Topical DOX (12), topical CIP (15)	Keratectomy	Failed
2	50	F	Trauma	Right hand	Suppurative granulomatous inflammation	DOX (7), CLR (21), CIP (21)	None	Failed
3	65	M	Fish tank	Right hand	Suppurative granulomatous inflammation	DOX (21), RIF (21), SXT (14)	None	Failed
4	69	F	Fish tank	Right hand	Granulomatous inflammation	DOX (18), SXT (18), CLR (35), CIP (143), RIF (187)	None	Relapse
5	31	F	ND	Right hand	Suppurative granulomatous inflammation	DOX (21)	None	Lost to follow-up
6	39	M	ND	Right forearm	Suppurative granulomatous inflammation	DOX (ND)	Debridement	Successful
7	41	M	Fish tank	Right hand	Suppurative granulomatous inflammation	DOX (28)	Debridement	Successful
8	51	M	Fish	Left hand	ND	DOX (73), RIF (220), EMB (220), CLR (147)	None	Successful

F: female, M: male, CIP: ciprofloxacin, CLR: clarithromycin, DOX: doxycycline, EMB: ethambutol, RIF: rifampicin, SXT: co-trimoxazole, ND: not documented.

## Discussion

Efficiently treatment of *M. marinum* infection remains a challenge. One of the reasons that make diagnosis difficult is the patients' lack of awareness coupled with the fact that *M. marinum* infections progress slowly, with a median (IQR) progression time of 3 (1–6) months. As the patients did not consult the doctor until the clinical symptoms worsened, it was difficult to identify the relationship between *M. marinum* skin infections and previous trauma history. Besides, cutaneous lesions are generally nonspecific and are often initially misdiagnosed as pyoderma, furunculosis, or even sporotrichosis [20]–[22]. Regarding the clinical treatment, *M. marinum* infections are usually treated empirically with antituberculosis agents, and antibiotic-susceptibility testing is not routinely performed in many clinical laboratories. These issues make the treatment of *M. marinum* even more difficult. The results of this study presented the antibiotic susceptibility pattern of *M. marinum* and characterized the relationship between antimicrobial therapy, surgery, and treatment outcome in *M. marinum* infections in Taiwan. These results may thus be used as a reference for clinical treatment of *M. marinum* infections in other countries.

The CLSI report does not recommend routine susceptibility testing of this species. However, the test may be necessary for some patients whose samples are still culture positive after receiving several months of unsuccessful therapy [23]. The results of *in vitro* susceptibility in this study showed a high susceptibility rate (>90%) for CLR, LZD, AMK, or SMX, which was consistent with effective treatment results. It is suggested that each single agent can be considered for treating in superficial *M. marinum* infections. From the viewpoint of pharmacokinetics, LZD could be a good alternative oral agent, although there have been no clinical trials till date to support this hypothesis [24]. Despite some previous reports showing successful treatment of *M. marinum* infection with DOX or minocycline [25], [26], the effectiveness of DOX treatment was still controversial due to several reports of treatment failure [11], [26]–[28]. Our results showed that *M. marinum* strains showed only 3.3% (1/30) sensitivity to DOX. This may reflect the high rate of treatment failure encountered in our patients treated with DOX. The controversy over DOX therapy may be related to the regional drug resistance pattern of *M. marinum*. Thus, it is suggested that drug susceptibility testing of *M. marinum* should be performed in patients whose treatment has failed and also in regions where the drug-resistance rate is high.

The potential reasons for the treatment failure/difficulty in *M. marinum* infections include delayed diagnosis and ineffective regimens in antimicrobial therapy. To date, there have been no comparative trials for treatment regimens in *M. marinum* skin and soft-tissue infections. A general approach is to treat patients with 2 active agents for 1–2 months after resolution of symptoms; the total treatment time is typically 3–4 months [9]. Furthermore, the duration of antimicrobial therapy in pulmonary or disseminated infections is also not standardized. Although, at present, there is no evidence indicating the superiority of combination therapy over monotherapy, there is a tendency to prescribe CLR plus EMB, or RIF plus EMB to treat a deep structure infection [9], [29]. In deep structure infections, especially on the hands, early diagnosis and appropriate therapy may play a key role in preventing the loss of normal function [30]. In addition, our results indicated surgical debridement may contribute to favorable treatment outcome.

Among *M. marinum* infections, a rare case involving a pulmonary infection was observed. The patient was a middle-aged woman and received interferon and ribavirin therapy for chronic hepatitis C. She then acquired interstitial pneumonitis where the *M. marinum* strain was isolated. This strain was sensitive to all tested antibiotics except DOX. Due to the delayed diagnosis and the lack of antimicrobial therapy, she developed sequelae such as pulmonary fibrosis and rheumatoid arthritis-like disorders. In 2005, Lai et al. also reported a case of pulmonary infection in an immunocompetent patient [6]. The 2 cases indicated *M. marinum* causes superficial infections, in addition to disseminated or even pulmonary infections. It would be worth monitoring and investigating this phenomenon closely for a longer duration.

In conclusion, optimal antimicrobial therapy consisting of CLR and/or RIF plus ethambutol, and surgical debridement may have a high treatment success rate in *M. marinum* infection. In contrast, DOX prescription is not suitable for this purpose.
